# Transcriptomic signatures reveal immune dysregulation in human diabetic and idiopathic gastroparesis

**DOI:** 10.1186/s12920-018-0379-1

**Published:** 2018-08-07

**Authors:** Madhusudan Grover, Simon J. Gibbons, Asha A. Nair, Cheryl E. Bernard, Adeel S. Zubair, Seth T. Eisenman, Laura A. Wilson, Laura Miriel, Pankaj J. Pasricha, Henry P. Parkman, Irene Sarosiek, Richard W. McCallum, Kenneth L. Koch, Thomas L. Abell, William J. Snape, Braden Kuo, Robert J. Shulman, Travis J. McKenzie, Todd A. Kellogg, Michael L. Kendrick, James Tonascia, Frank A. Hamilton, Gianrico Farrugia, the NIDDK Gastroparesis Clinical Research Consortium (GpCRC), Jose Serrano, Linda Nguyen, William Hasler, Kristy Zodrow

**Affiliations:** 1 Enteric NeuroScience Program, Division of Gastroenterology & Hepatology, Mayo Clinic, 200 1st Street SW, Rochester, MN 55905 USA; 2 Biomedical Statistics and Informatics, Mayo Clinic, Rochester, MN USA; 3 Johns Hopkins University Bloomberg School of Public Health, Johns Hopkins University, Baltimore, MD USA; 4 Johns Hopkins University School of Medicine, Baltimore, MD USA; 5 Temple University, Philadelphia, PA USA; 6 Texas Tech University, El Paso, TX USA; 7 Wake Forest University, Winston-Salem, NC USA; 8 University of Louisville, Louisville, KY USA; 9 California Pacific Medical Center, San Francisco, CA USA; 10 Massachusetts General Hospital, Boston, MA USA; 11 Baylor College of Medicine, Houston, TX USA; 12 Department of Surgery, Mayo Clinic, Rochester, MN USA; 13 National Institute of Diabetes and Digestive and Kidney Diseases, Bethesda, MD USA

**Keywords:** Diabetes mellitus, Next generation sequencing, Macrophages, RNA, Signaling

## Abstract

**Background:**

Cellular changes described in human gastroparesis have revealed a role for immune dysregulation, however, a mechanistic understanding of human gastroparesis and the signaling pathways involved are still unclear.

**Methods:**

Diabetic gastroparetics, diabetic non-gastroparetic controls, idiopathic gastroparetics and non-diabetic non-gastroparetic controls underwent full-thickness gastric body biopsies. Deep RNA sequencing was performed and pathway analysis of differentially expressed transcripts was done using Ingenuity®. A subset of differentially expressed genes in diabetic gastroparesis was validated in a separate cohort using QT-PCR.

**Results:**

111 genes were differentially expressed in diabetic gastroparesis and 181 in idiopathic gastroparesis with a log_2_fold difference of | ≥ 2| and false detection rate (FDR) < 5%. Top canonical pathways in diabetic gastroparesis included genes involved with macrophages, fibroblasts and endothelial cells in rheumatoid arthritis, osteoarthritis pathway and differential regulation of cytokine production in macrophages and T helper cells by IL-17A and IL-17F. Top canonical pathways in idiopathic gastroparesis included genes involved in granulocyte adhesion and diapedesis, agranulocyte adhesion and diapedesis, and role of macrophages, fibroblasts and endothelial cells in rheumatoid arthritis. Sixty-five differentially expressed genes (log_2_fold difference | ≥ 2|, FDR < 5%) were common in both diabetic and idiopathic gastroparesis with genes in the top 5 canonical pathways associated with immune signaling. 4/5 highly differentially expressed genes (SGK1, APOLD1, CXCR4, CXCL2, and FOS) in diabetic gastroparesis were validated in a separate cohort of patients using RT-PCR. Immune profile analysis revealed that genes associated with M1 (pro inflammatory) macrophages were enriched in tissues from idiopathic gastroparesis tissues compared to controls (*p* < 0.05).

**Conclusions:**

Diabetic and idiopathic gastroparesis have both unique and overlapping transcriptomic signatures. Innate immune signaling likely plays a central role in pathogenesis of human gastroparesis.

**Electronic supplementary material:**

The online version of this article (10.1186/s12920-018-0379-1) contains supplementary material, which is available to authorized users.

## Background

Diabetic and idiopathic gastroparesis result in significant morbidity and health-care utilization [1]. There is an unmet need for safe and efficacious treatment options [2]. Our mechanistic understanding of gastroparesis has expanded in the last decade; however, actionable therapeutic targets are still missing. We and others have shown that loss of interstitial cells of Cajal (ICC) is a primary cellular injury in animal models and in patients with gastroparesis [3, 4]. In diabetic gastroparesis, this decrease in ICC number correlates with the degree of gastric retention [5]. Ultrastructural studies have allowed us to examine defects in ICC and the broader neuromuscular apparatus [6]. Diabetic gastroparetics had thickened basal lamina around smooth muscles and nerves, whereas, idiopathic gastroparetics had fibrosis, especially around the nerves. This suggests possibility of both overlapping and unique mechanistic aspects for diabetic and idiopathic gastroparesis.

Recent animal studies have provided a paradigm for immune mediated injury of the enteric neuromuscular apparatus, including ICC, to be central in pathogenesis of gastroparesis. In the non-obese diabetic (NOD) mouse model, the proportion of CD206, heme oxygenase-1 (HO1) –positive “M2” macrophages (alternatively activated or anti-inflammatory) increased with development of diabetes. However, the development of delayed gastric emptying was associated with an increase in iNOS expression, a marker for macrophages with a “M1” phenotype (classically activated or pro-inflammatory) [7]. Macrophage-deficient CSF1^op/op^ mice did not develop delayed gastric emptying despite severe diabetes [8]. Decreased CD206^+^ (M2 macrophages) have also been reported in the gastric antrum of patients with diabetic and idiopathic gastroparesis which correlated with the loss of ICC [9]. Additionally, HO1 expressed in M2 macrophages is directly associated with delayed gastric emptying in diabetic mice and polymorphisms in the HO1 gene (HMOX1) are associated with worse outcomes in human diseases [7].

Despite the advances and insights gained from animal models and human studies, our mechanistic understanding of physiological and clinical changes in human gastroparesis is still limited. The NIDDK Gastroparesis Clinical Research Consortium (GpCRC) collects full thickness gastric tissue from patients with diabetic and idiopathic gastroparesis. We undertook a hypothesis generating approach to attempt to obtain molecular and cellular targets for future mechanistic and therapeutic studies. Our aim was to determine the abundance and relationships between gene transcripts in diabetic and idiopathic gastroparesis by deep sequencing of RNA extracted from the smooth muscle layers including the myenteric plexus of the human gastric body. Secondly, we aimed to identify transcription-based signaling pathways in diabetic and idiopathic gastroparesis.

## Methods

### Specimens

Full thickness gastric body biopsies were obtained from 7 diabetic gastroparetics, 7 diabetic non-gastroparetic controls, 5 idiopathic gastroparetics and 7 non-diabetic non-gastroparetic controls. Differences between female and male transcriptome are expected and the prevalence of gastroparesis is higher in females. Hence, in this pilot study, only female transcriptome was determined to allow sufficient power for analysis for one sex. The gastroparesis patients were undergoing implantation of a gastric electrical stimulator at the time of tissue procurement, and the controls were undergoing obesity surgery. 37% of gastroparetic patients were obese (BMI > 30 kg/m^2^). All patients were > 18 years of age with symptoms of at least 12-weeks duration, delayed gastric emptying on scintigraphy (> 60% retention at 2 h or > 10% retention at 4 h), and no evidence of gastric outlet obstruction. Exclusion criteria included presence of active inflammatory bowel disease, eosinophilic gastroenteritis, neurological conditions, acute liver or renal failure, and history of total or subtotal gastric resection. Tissue collection was done in standardized fashion from the gastric body with established protocols by the participating sites of the GpCRC. As a part of this, the surgeon is required to follow an exact protocol for procuring tissue from a precisely defined location and mark the position on a working sheet. Following this, a member of the research team ensures that the biopsies are promptly preserved in appropriate solutions. The pathology core at Mayo Clinic prepares and ships fixative solutions to all sites. The tissue is shipped as overnight priority to Mayo Clinic and is subsequently processed using standardized procedures. The mucosa was peeled and the muscularis sample was cryopreserved in RNAlater until further use. All gastroparesis patients provided written informed consent for procurement and use of gastric tissue at the clinical sites of GpCRC. All clinical sites of GpCRC had approval from their institutional IRBs. All control tissues were obtained in a de-identified fashion at Mayo Clinic in an IRB-approved protocol. Oral consent was obtained for use of this tissue.

### RNA extraction

Total RNA was isolated from the smooth muscle using RNA-Bee (TelTest, Friendswood, TX) and purified for sequencing using the RNeasy Plus Mini Kit (Qiagen, Valencia CA). Samples were tested by the Mayo Gene Expression Core using the Agilent Bioanalyzer and all had a RNA Integrity Numbers (RIN) > 7.0.

### RNA sequencing

Samples were sequenced using the Illumina TruSeq v2 library prep kit with 3 samples in each lane using a paired end index read with 51 base reads. RNA-Seq data were analyzed by the Mayo Clinic Division of Biomedical Statistics and Informatics using a comprehensive bioinformatics pipeline, defined MAP-RSeq (version 2.1.1), a streamlined pipeline for processing paired-end RNA sequencing reads with low intervention during the analysis stage [10]. The MAP-RSeq utilized a variety of freely available bioinformatics tools along with in-house developed methods to align, assess and provide multiple genomic features from transcriptomic sequencing data for further downstream analysis.

### Differential expression analysis

The data were subjected to principal component analysis (PCA) using the Partek Suite to establish whether the samples in each group had differing expression profiles. Differentially expressed genes were determined using the edgeR package [11]. The criterion for considering a gene transcript to be differentially expressed was having an adjusted probability of significance (adjusted P) less than 0.05 (or false discovery rate, FDR < 5%). Separately, transcripts with a log_2_ fold difference of 2 and higher or − 2 and lower (log_2_FC |2|) were used for creating heat maps and for pathway analysis. We used R packages, edgeR [11] and RNASeqPower [12] to obtain coefficient of variation and perform statistical power analysis. In our cohort, 90% of the expressed genes have a coefficient of variation less than 0.42. Using the formula from RNASeqPower, setting the type I error to 0.05 and the power to 80%, 13 samples per group would be needed to observe a 2-fold expression in a gene. Seven subjects per group provide 51% power to detect a 2-fold expression in a gene.

### Pathway analysis

Ingenuity pathway analysis (IPA) was used to determine pathways and other associations connecting the differentially expressed transcripts [13].

### Immune profile analysis

To determine the immune cell composition of the gastroparesis and control RNA samples, normalized gene counts (reads per kilobase per million mapped reads, RPKM) generated from MAP-RSeq were subset to the LM22 signature gene set from CIBERSORT [14]. The probability values reported by CIBERSORT for M1 and M2 macrophages were assessed for differences between the diabetic and idiopathic gastroparesis groups compared to their controls using a 2-sided non-parametric unpaired t-test. A *p* < 0.05 was considered statistically significant.

### RT-PCR for validation

Transcripts that are altered in both diabetic and idiopathic gastroparesis were determined. Subsequently, genes with putative biological significance for gastroparesis were identified and their expression was determined in a separate validation cohort of full thickness gastric body tissue from 6 diabetic gastroparesis subjects and 6 diabetic controls using RT PCR. β-actin was used as the housekeeping gene and delta Ct values were calculated. The following genes were sequenced: SGK, serum/glucocorticoid regulated kinase 1; APOLD1, apolipoprotein L domain containing 1; CXCR4, C-X-C motif chemokine receptor 4; CXCL2, C-X-C motif chemokine ligand 2; and FOS, Fos proto-oncogene.

## Results

### Patient characteristics

Table 1 highlights the demographic and disease characteristics of diabetic gastroparetics, diabetic non-gastroparetic controls, idiopathic gastroparetics and non-diabetic non-gastroparetic controls. The median age was similar among the 4 groups. The overall gastroparesis cardinal symptom index (GCSI) score and the subtype scores (nausea, fullness and bloating) were similar between diabetic and idiopathic gastroparetics*.*

**Table 1:** Demographic and disease characteristics of the gastroparesis patients (diabetic, idiopathic) and controls (diabetic, non-diabetic)

	Diabetic Gastroparesis	Diabetic Control	Idiopathic Gastroparesis	Non-diabetic Control
Age (median, range)	39; 24–59	46; 33–57	40; 26–64	39; 26–48
Diabetes, Type	I: 6; II: 1	–	–	–
% Gastric emptying				
2 h	46.2 (25.6)	–	41.5 (8.7)	–
4 h	36.5 (30)	–	18.7 (11.9)	–
GCSI, overall	3.7 (0.9)	–	3.5 (0.7)	–
Nausea	3.5 (1.5)	–	2.8 (1.7)	–
Fullness	4.2 (0.6)	–	3.5 (0.3)	–
Bloating	3.5 (1.3)	–	4.1 (0.6)	–

### Sequencing depth

RNA samples had a minimum RIN value of 7.4 and at least 80 million reads were obtained from each sample (Additional file 1: Table S1). At least 60,000,000 reads mapped to 64,253 identified gene transcripts. The gene annotation used was from Ensembl release 78. The transcript identifiers were obtained from the gene definition file for *Homo sapiens* GRCh38.78, obtained from the Ensembl ftp server (ftp://ftp.ensembl.org/pub/release-78/gtf/homo_sapiens/). Samples were enriched for markers of smooth muscle (ACTG2, ACTB, ACTA2), neurons (UCHL1), glia (S100B) and, to a lesser extent, ICC (Kit, Ano1) indicating that the tissue was enriched for cells from the muscularis propria of the gastric wall. Minimal contamination with RNA derived from the mucosa was detected as indicated by low numbers of reads for genes enriched in the mucosal layers of the GI including trefoil factors (TFF1 and TFF2), gastric lipase (LIPF), gastric intrinsic factor (GIF) and pepsinogens (PGA3 and PGA5). The dataset has been submitted to Gene Expression Omnibus (GSE115601).

### Differentially expressed genes in diabetic gastroparesis

**Fig. 1:**
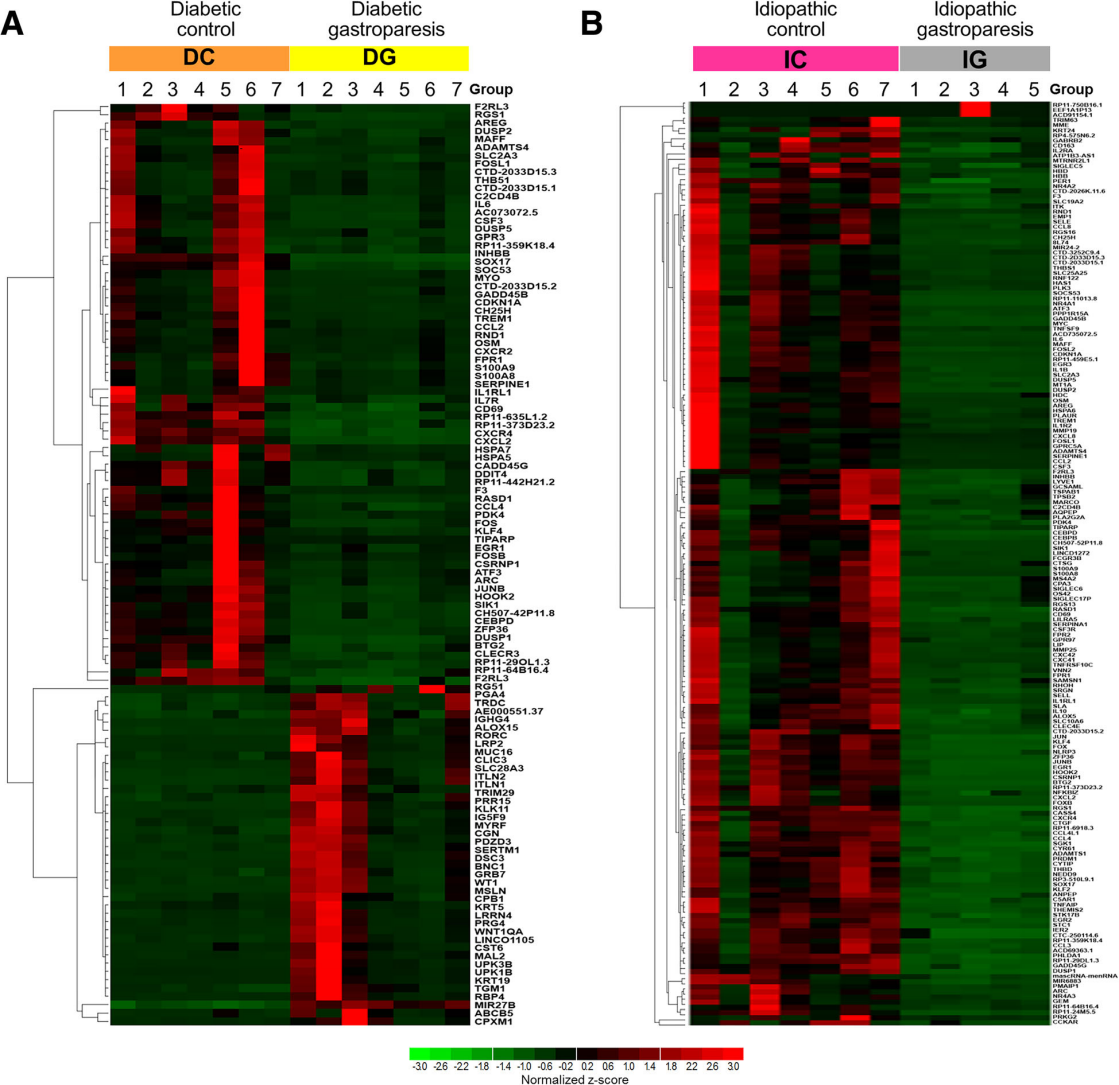
Heat maps of differentially expressed genes (Log_2_fold change | ≥ 2|, FDR < 0.05) in (**a**) Diabetic gastroparesis and (**b**) Idiopathic gastroparesis

Three hundred and seventy-three gene transcripts were differentially expressed (FDR < 5%) among diabetic gastroparetics and diabetic non-gastroparetic controls (Additional file 2: Table S2). 104 of the 373 were upregulated and the remaining genes were downregulated in diabetic gastroparesis. Expression levels in RPKM for one hundred and eleven genes with a log_2_fold difference in expression of | ≥ 2| as compared to diabetic controls are shown in the heatmap (Fig. 1a). When diabetic gastroparetics were compared with non-diabetic controls, 568 genes were differentially expressed (Additional file 3: Table S3); 130 of those had a log_2_fold difference in expression of | ≥ 2|.

Genes with log_2_fold difference in expression of | ≥ 2| (*n* = 111) were analyzed using the Ingenuity pathway analysis. The 3 top canonical pathways were associated with role of macrophages, fibroblasts and endothelial cells in rheumatoid arthritis (*p*-value 1.12E-07), osteoarthritis pathway (*p*-value 3.63E-07) and differential regulation of cytokine production in macrophages and T helper cells by IL-17A and IL-17F (*p*-value 9.74E-07). However, only 3–22% differentially expressed genes in our dataset overlapped with the total genes known to be associated with these pathways in the Ingenuity database. Four genes that were common to at least 2/3 top canonical pathways were CCL2, IL6, IL1RL1 and ADAMTS4, all downregulated in diabetic gastroparesis. Hematological system development and function was the leading physiological function identified linked by 49 of the 130 molecules with quantity of leucocytes as the key annotated function associated with 34 of those molecules (Increased ALOX15 and RORC; Decreased ATF3, CCL2, CCL4, CD69, CDKN1A, CLEC4E, CSF3, CXCL2, CXCR2, CXCR4, DUSP1, DUSP5, EGR1, F3, FOS, FOSB, FPR1, GADD45B, GADD45G, IL1RL1, IL6, IL17R, KLF4, MYC, OSM, RGS1, S100A8, S100A9, SERPINE1, SOCS3, THBS1, and ZFP36). The top biological gene interaction containing the most number of differentially expressed genes in our group was “inflammatory disease, cellular movement, hematological system development and function”.

### Differentially expressed genes in idiopathic gastroparesis

Seven hundred and twenty-seven gene transcripts were differentially expressed between idiopathic gastroparetics and non-diabetic non-gastroparetic controls (Additional file 4: Table S4). Of those, 73 genes were upregulated in idiopathic gastroparesis and the remaining genes were downregulated. Expression levels in RPKM are shown for one hundred eighty-one genes with at least a | ≥ 2| log_2_fold difference in expression as compared to controls are shown in the heatmap (Fig. 1b).

Genes with log_2_fold difference (*n* = 181) in expression of | ≥ 2| were analyzed using the Ingenuity pathway analysis. This revealed the 3 top canonical pathways with genes associated with granulocyte adhesion and diapedesis (*p*-value 3.49E-20), agranulocyte adhesion and diapedesis (*p*-value 2.68E-13), and role of macrophages, fibroblasts and endothelial cells in rheumatoid arthritis (*p*-value 4.74E-12). All of these genes were downregulated in idiopathic gastroparesis. Five genes that were common to all three top canonical pathways were C5AR1, CCL2, CXCL8, IL1B and SELE. There was 6–12% overlap in genes associated with these functions in these top pathways and the differentially expressed genes in the Ingenuity dataset. A fourth canonical pathway contained 7 of the 18 genes (CCL2, CCL3, CCL4, CSF3, IL6, IL10 and IL1B) that have been associated with differential regulation of cytokine production in macrophages and T helper cells by IL-17A and IL-17F. Immune cell trafficking was the leading physiological function identified linked by 82 of the 181 molecules with leucocyte migration as the key annotated function associated with 71 of those molecules. The top biological gene interaction network containing the most number of differentially expressed genes in our group was “cellular movement, immune cell trafficking, cell-to-cell signaling and interaction”.

### Differentially expressed genes common to diabetic and idiopathic gastroparesis

**Fig. 2:**
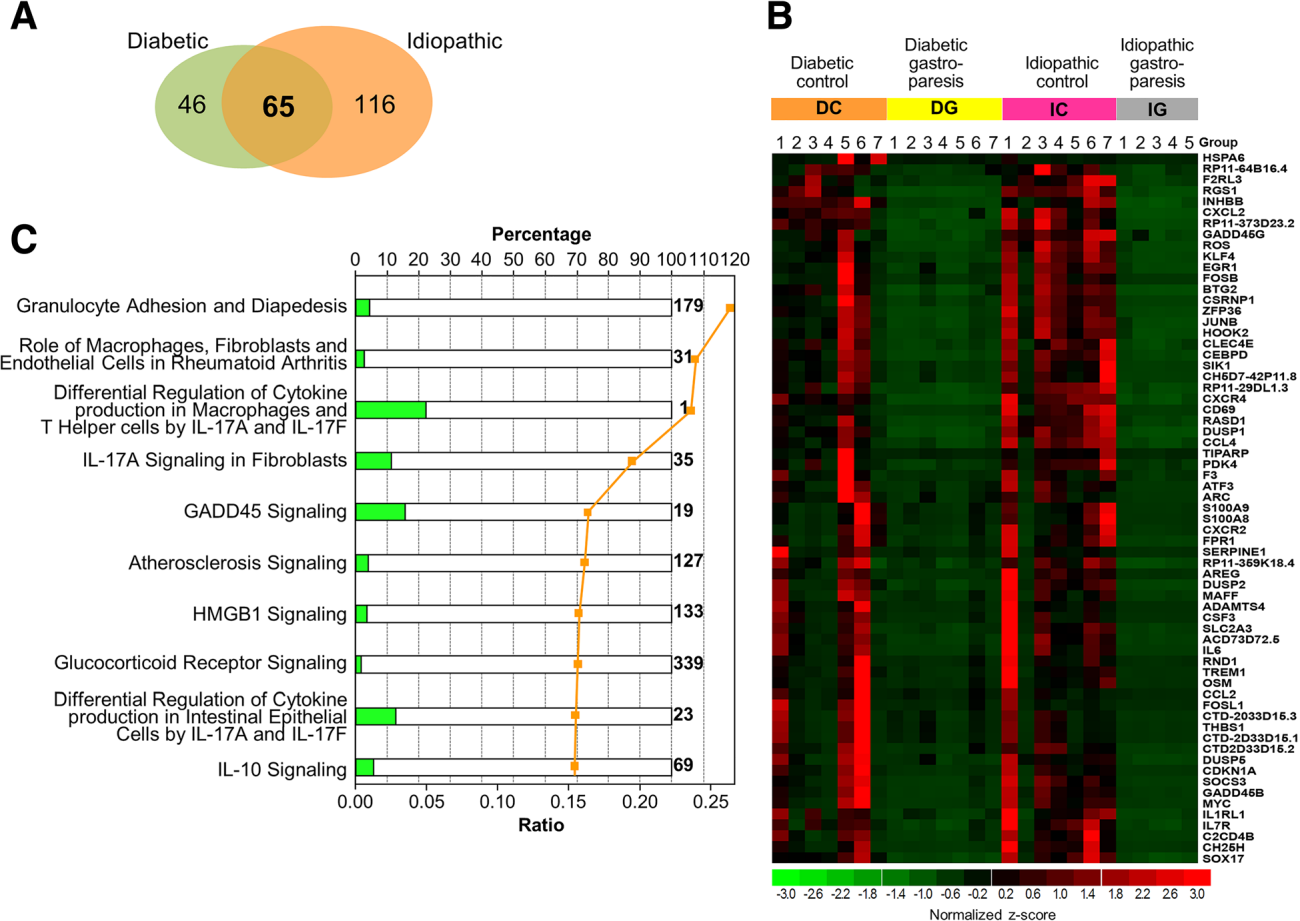
**a** Venn diagram showing overlapping differentially expressed genes between diabetic gastroparetics and diabetic controls and idiopathic gastroparetics and idiopathic controls (Log_2_fold change | ≥ 2|, FDR < 0.05). **b** Heat map demonstrating these overlapping, differentially expressed genes between diabetic gastroparetics and diabetic controls and idiopathic gastroparetics and idiopathic controls (Log2fold change | ≥ 2|, FDR < 0.05). **c** Top canonical pathways linking the overlapping genes involved between the two comparisons. The horizontal bars represent total number of genes present in the pathway, scaled to 100%. The orange dots indicate the ratio of the overlapping differentially expressed genes that map to the pathway divided by the total number of genes present in the same pathway, e.g. > 25% for Granulocyte Adhesion and Diapedesis

Two hundred genes were differentially expressed in both diabetic and idiopathic gastroparesis as compared to their controls (Additional file 5: Table S5). Distribution of common and overlapping genes between the two comparison groups with a log_2_fold difference of | ≥ 2| is shown in Fig. 2a. Sixty five genes were common to both groups and all of them were downregulated in gastroparesis (Fig. 2b). The top 3 canonical pathways had genes associated with granulocyte adhesion and diapedesis (CCL2, CCL4, CSF3, CXCL2, CXCR2, CXCR4, FPR1, and IL1R1, *p* = 1.74E-08), role of macrophages, fibroblasts and endothelial cells in rheumatoid arthritis (ADAMTS4, CCL2, CEBPD, FOS, IL6, IL1R1, MYC, OSM, and SOCS3, *p* = 9.13E-08), and differential regulation of cytokine production in macrophages and T helper cells by IL-17A and IL-17F (CCL2, CCL4, CSF3 and IL6, *p* = 1.15E-07) (Fig. 2c). The differential regulation of cytokine production in macrophages and T helper cells by IL-17A and IL-17F had 4 genes overlapping with 18 associated with that pathway (22% overlap). Hematological system development and function (41 genes) and tissue morphology (38 genes) were the two top physiological systems affected with the function “quantity of leucocytes” linking most differentially expressed genes.

### RT-PCR validation of common genes

**Fig. 3:**
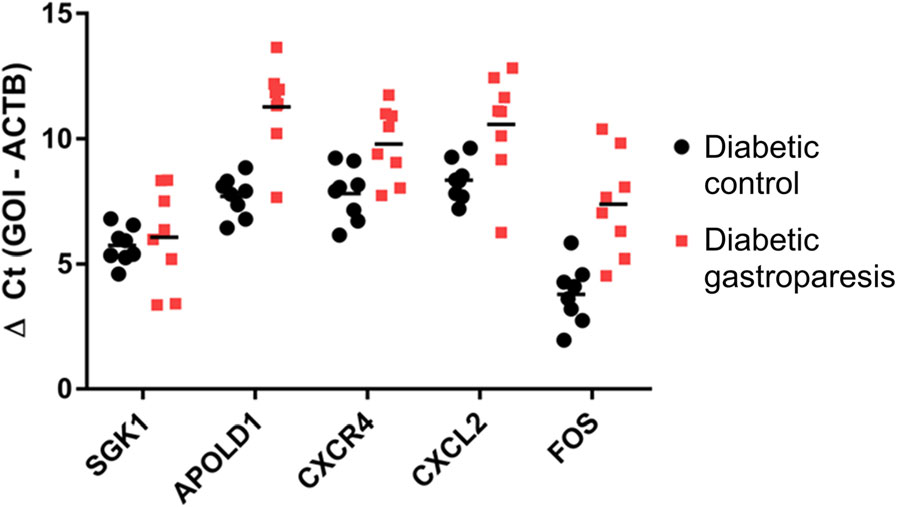
RT-PCR validation of 5 genes differentially expressed in diabetic gastroparetics and diabetic controls by RNA seq in a different set of diabetic gastroparesis patients: Significant downregulation of APOLD1, apolipoprotein L domain containing 1; CXCR4, C-X-C motif chemokine receptor 4; CXCL2, C-X-C motif chemokine ligand 2; and FOS, Fos proto-oncogene in diabetic gastroparesis, as observed in the RNA seq analysis. SGK1, serum/glucocorticoid regulated kinase 1 expression was not statistically different on RT-PCR in the validation cohort, but had significantly lower log2fold changes in expression on RNA seq (1.77)

Five highly differentially expressed genes in diabetic gastroparesis were selected for validation studies (SGK1, serum/glucocorticoid regulated kinase 1; APOLD1, apolipoprotein L domain containing 1; CXCR4, C-X-C motif chemokine receptor 4; CXCL2, C-X-C motif chemokine ligand 2; and FOS, Fos proto-oncogene). Four of these were significantly downregulated in diabetic gastroparesis (APOLD1, CXCR4, CXCL2, and FOS) as observed in the RNA seq analysis (Fig. 3). The one gene that was not found to be statistically different on RT-PCR had significant but lower log_2_fold changes in expression on RNA seq (SGK1–1.77). The most robust differences were seen in APOLD1, apolipoprotein L domain containing 1 (ΔΔCT = 3.595, *P* < 0.0001) and FOS, Fos proto-oncogene (ΔΔCT = 3.588, P < 0.0001).

### Immune profile analysis

**Fig. 4:**
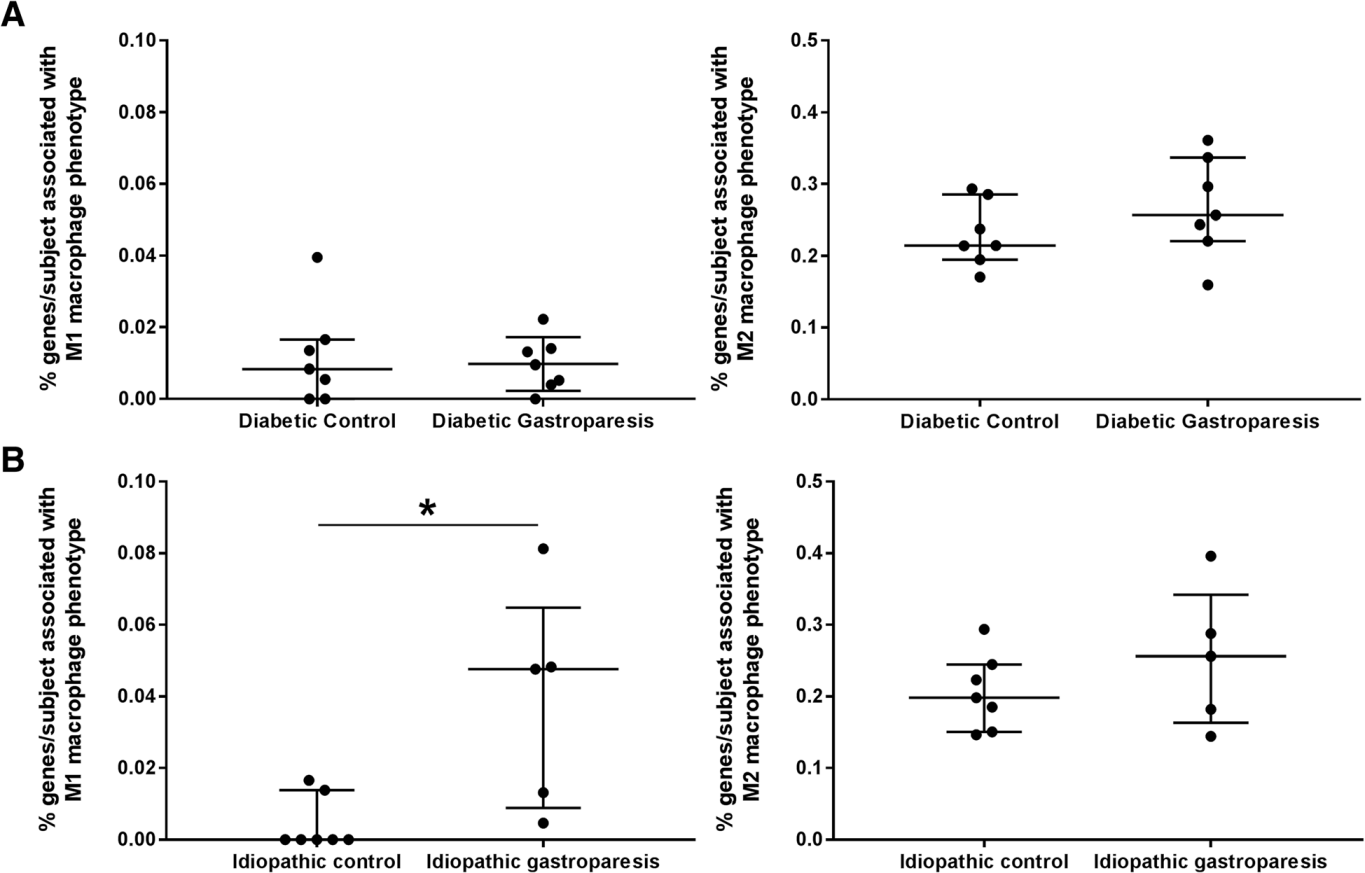
CIBERSORT analysis displaying distribution of patients with M1 and M2 macrophage associated genes in (**a**) Diabetic controls and diabetic gastroparesis: No differences were seen in % genes/subject associated with M1 or M2 macrophage phenotype (**b**) Idiopathic controls and idiopathic gastroparesis: Significantly greater number of idiopathic gastroparesis patients express % genes associated with an M1 (proinflammatory) macrophage phenotype (Mean (SD): 0.04 (0.03) % vs 0.004 (0.007) % M1 associated genes in idiopathic gastroparesis and idiopathic controls respectively, *p* = 0.02). No differences were seen in % genes/subject associated with M2 macrophage phenotype

The CIBERSORT immune cell analysis revealed no significant differences in enrichment of M1 or M2 macrophage associated genes in the diabetic gastroparesis and diabetic control samples. However, M1 (pro-inflammatory) macrophage associated genes were significantly more highly represented in idiopathic gastroparetic samples than in non-diabetic, non-gastroparetic controls (Mean (SD): 0.04 (0.03) % vs 0.004 (0.007) % M1 associated genes in idiopathic gastroparesis and idiopathic controls respectively, *p* = 0.02). The quantification for percentage of genes associated with these macrophage subtypes in diabetic and idiopathic gastroparesis vs their controls are displayed in Fig. 4.

## Discussion

Recent advancements made in molecular understanding of gastroparesis using animal models and human samples have elucidated an important role of the innate immune system [15]. This is likely driven by a shift in polarization of macrophages and associated expression of pro- and anti-inflammatory cytokines. The current proposed central mechanism for gastroparesis includes macrophage driven loss of or functional abnormalities in ICC and other components of the enteric neuromuscular apparatus which can then affect gastric motility.

This study utilizing deep transcriptional sequencing of the human stomach neuromuscular apparatus further provides evidence for innate immune dysregulation as the key feature of both diabetic and idiopathic gastroparesis. More specifically, it highlights that macrophage function and signaling is associated with this disease process. Additionally, this study identifies pathways that are potentially unique to diabetic and idiopathic gastroparesis. Macrophage and T helper cell signaling genes were differentially expressed in diabetic gastroparesis, whereas, granulocyte and agranulocyte function genes were associated with pathways involved in idiopathic gastroparesis.

This study builds upon findings from mouse models and in vitro studies. In the NOD mouse model of gastroparesis, animals that retain normal gastric emptying have normal ICC networks and CD206-positive, HO1-positive M2 macrophages (anti-inflammatory spectrum) in the muscularis propria, while animals developing delayed gastric emptying demonstrated damage in ICC networks and express CD206-negative and HO1-negative M1 macrophages (pro-inflammatory spectrum) [7]. Furthermore, the presence of macrophages was found to be essential for development of delayed gastric emptying [8]. Our in vitro work has also provided evidence for M1 macrophage generated TNF-α to be involved in caspase-mediated apoptosis and Kit down regulation in ICC [16]. Finally, human studies have shown a positive correlation between the number of ICC and M2 macrophages in diabetics and diabetic gastroparesis [17]. The loss of ICC has been correlated with impairment in gastric emptying [5].

A large number of transcripts were found to be significantly altered in both diabetic and idiopathic gastroparesis patients compared to their controls. Four transcripts common to top canonical pathways in diabetic gastroparesis include CCL2 (C-C Motif Chemokine Ligand 2), a monocyte chemoattractant protein coding gene; IL6, pro-inflammatory gene involved in differentiation of B-cells, monocyte and generation of Th17 cells and also involved in improving insulin resistance and susceptibility to diabetes mellitus; IL1RL1 (Interleukin 1 Receptor Like 1), a receptor involved in proinflammatory stimuli and function of T helper cells; and ADAMTS4 (ADAM Metallopeptidase with Thrombospondin Type 1 Motif 4), a protease involved in degradation of cartilage protein aggrecan and extracellular matrix protein brevican. All of these transcripts were downregulated in diabetic gastroparesis. In idiopathic gastroparesis, five transcripts common to all three top canonical pathways include C5AR1 (Complement C5a Receptor 1), receptor for inflammatory peptide C5a involved in both innate and adaptive immune response; CCL2 (C-C Motif Chemokine Ligand 2); CXCL8 (C-X-C Motif Chemokine Ligand 8) chemotactic factor for neutrophils, basophils, and T-cells; IL1B (Interleukin 1 Beta), a macrophage produced cytokine involved in acute B and T cell activation and cyclooxygenase 2 activation; and SELE (Selectin E), a leucocyte cell surface adhesion protein. All of these transcripts were downregulated in gastroparesis. It remains to be further investigated whether these changes have an influence on the protein expression and it should be kept in mind that the majority of differentially expressed genes in our dataset did not overlap with the total genes known to be associated with these pathways in the Ingenuity database suggesting that the specific pathways involved may be different. When transcripts common to diabetic and idiopathic gastroparesis were analyzed, five transcripts associated with IL-17A signaling (CCL2, CEBPD, FOS, IL6 and JUN) were found to be involved. IL-17A is a pro-inflammatory cytokine that regulates NF-kappa B and mitogen-activated protein kinases and can stimulate expression of IL6 and COX-2 [18, 19]. It has been shown to stimulate inflammatory activity in human subepithelial myofibroblasts [20] and involved in and production of nitric oxide in endothelial cells [21].

Of interest is the significant overlap of genes between diabetic and idiopathic gastroparesis that was found in this study. There currently is a healthy and vigorous debate on whether idiopathic gastroparesis belongs more to the functional bowel disorder spectrum of diseases or more to the diabetic gastroparesis spectrum. The findings of this study suggest similarities in pathophysiology between diabetic and idiopathic gastroparesis and presents mechanistic insight into idiopathic gastroparesis as a non-functional disease. Interestingly, almost twice the numbers of transcripts were altered in idiopathic gastroparesis compared to diabetic gastroparesis when compared with their controls.

The CIBERSORT analysis provided a complementary approach at determining associations between abundance of specific cell type associated genes in specific subjects within the disease groups. This showed a higher abundance of genes indicating presence of M1 (pro-inflammatory spectrum) macrophages in the idiopathic gastroparesis tissues when compared to their controls. This is interesting since in in vitro studies, M1 macrophage derived TNFα was found to cause loss of ICC through reduced Kit expression [16].

Significant challenges exist in the studies of human macrophages due to diversity of macrophage phenotypes and considerable differences between markers for mouse and human macrophages. In mice, mucosal monocytes with lymphocyte antigen 6C (Ly6C) expression differentiate into pro-inflammatory (M1) and inflammatory dendritic cells upon entering the tissue [22]. However, in humans Gr1+, an equivalent of mouse Ly6C is not associated with expression of proinflammatory markers [23]. In lung tissue, TGM2 was found to be a robust marker for the anti-inflammatory macrophage subtype; however, this has not been validated in gastrointestinal tissues [24]. The marker used to study macrophage subtypes depend upon disease, species and sites involved. In general, the dichotomous differentiation of M1 and M2 phenotype is currently discouraged and these phenotypes should be seen in a spectrum. iNOS is expressed robustly in mouse macrophages in inflammation but is repressed epigenetically in human macrophages [25]. Arginase-1 and Ym1 are examples of other proteins that are expressed in mouse but not human M2 macrophages [26]. Therefore, classification of human macrophages continues to be a work in progress. In both mice and human, muscularis macrophages have been identified in close proximity to ICC suggesting a role in their function [27]. A variety of neurotransmitter receptors on macrophages regulate their functional phenotype. Additionally, neurons express receptors for macrophage-derived signaling molecules that alter neuronal activity and survival including bone morphogenetic protein 2 [28] and TNFα [29]. Nitric oxide produced by iNOS in mouse (but not human) macrophages suppresses neuronal [30] and smooth muscle excitability [31]. A recent study of idiopathic gastroparetics revealed decreased expression of PDGFRα and its ligands PDGFA and PDGFB, smooth muscle proteins (MYH11, MYLK1, and PAK1), non-specific macrophage marker (CD68), gene encoding HO1 (HMOX1) and immunosuppressive cytokine TGFβ1 [32]. Functional interactions between macrophages and ICC and other components of neuromuscular apparatus in humans need to be studied.

A limitation of the study is the small sample size. Seven subjects per group provide 51% power to detect a 2-fold expression in a gene, indicating that certain important genes may have been missed. These findings will require validation in a larger cohort of gastroparesis patients. This will also be necessary to determine if there are subsets within gastroparesis with distinct transcriptional profiles based on symptoms, gastric emptying delay or overlap with other diagnoses. Secondly, all controls were obese, whereas, only 37% of gastroparesis patients were obese. Since obesity is also a proinflammatory state, this could have confounded the results of the analysis. However, in our previous study no differences in global immune cell population (CD45 immunoreactive cells) were seen between obese and non-obese subjects [3]. Third, the databases used for Ingenuity pathway analysis are enriched in transcripts associated with immune and cancer biology. This may skew pathways analyses towards those biological functions. Another observation from the current study is lack of evidence of pathways linking transcripts for neuromuscular function which synchronizes with our findings at protein level, where nerve and neuronal changes were seen in only a subset of patients with gastroparesis. Although, this may be due to limitations of the Ingenuity platform, another possibility includes that cumulative transcriptional expression in a complex biological sample containing multiple cell types may mask changes seen in gene expression in specific cell-types.

## Conclusions

This study is the first to determine whole transcriptomic changes in deeper neuromuscular apparatus in gastroparesis (or any gastrointestinal motility disorder). Several important observations are made. First, diabetic and idiopathic gastroparesis have both unique and overlapping changes in the transcriptome. However, immune signaling predominates the pathways linking most of the differentially expressed transcripts. Second, most patients with idiopathic gastroparesis differentially expressed transcripts compared to controls that have been associated with pro-inflammatory (M1) macrophage phenotype. Third, transcriptomics provides reliable targets for future mechanistic work considering the validation of selected genes achieved in a separate set of diabetic gastroparesis patients. These findings make a case for targeting innate immune system for development of future treatment approaches to gastroparesis but the molecular targets identified will need further investigations in animal models to determine the optimal timing of intervention aimed at preventing or reversing damage to the gastric function.

## Additional files

Additional file 1: **Table S1.** RNA reads per sample. (DOCX 16 kb)

Additional file 2: **Table S2.** Differentially expressed genes in diabetic gastroparesis and diabetic controls. (XLSX 93 kb)

Additional file 3: **Table S3.** Differentially expressed genes in diabetic gastroparesis and non-diabetic controls. (XLSX 127 kb)

Additional file 4: **Table S4.** Differentially expressed genes in idiopathic gastroparesis and idiopathic controls. (XLSX 155 kb)

Additional file 5: **Table S5.** Differentially expressed genes common in diabetic gastroparesis and diabetic controls and idiopathic gastroparesis and controls. (XLSX 107 kb)

## Abbreviations

GI: Gastrointestinal tract
HO1: Heme oxygenase 1
ICC: Interstitial cells of Cajal
IL10: Interleukin 10
IL6: Interleukin 6
iNOS: Inducible NOS
NOD: Non-obese diabetic
op/op: Osteopetrotic mouse
TNFα: Tumor necrosis factor-α
