# Supplementary material for: Transcriptomic signatures reveal immune dysregulation in human diabetic and idiopathic gastroparesis
Source: BMC Med Genomics. 2018 Aug 7;11:62. doi: 10.1186/s12920-018-0379-1 (PMC6081936; doi:10.1186/s12920-018-0379-1)
Supplement: Supplementary file 1 — Table S1. RNA reads per sample. (DOCX 16 kb) [file 12920_2018_379_MOESM1_ESM.docx]

**Additional Table 1**: Sequencing depth in individual patient samples

| **Diabetic gastroparetics** | | | | |
| --- | --- | --- | --- | --- |
|  | **RIN** | **Total reads** | **Mapped Reads** | **Gene Count** |
| Patient 1 | 8.6 | 124,782,988 | 120,318,478 | 92,582,474 |
| Patient 2 | 8.4 | 105,241,490 | 102,676,780 | 83,035,434 |
| Patient 3 | 8.6 | 108,912,350 | 105,530,676 | 80,881,864 |
| Patient 4 | 7.6 | 83,471,742 | 80,917,314 | 61,299,812 |
| Patient 5 | 8.3 | 131,813,428 | 122,606,165 | 95,867,020 |
| Patient 6 | 7.9 | 112,293,398 | 102,545,617 | 75,994,494 |
| Patient 7 | 9.0 | 130,865,492 | 127,443,685 | 99,831,688 |
| **Diabetic controls** | | | | |
| Control 1 | 8.9 | 126,799,132 | 120,411,767 | 92,476,396 |
| Control 2 | 7.9 | 123,690,994 | 116,250,805 | 88,950,992 |
| Control 3 | 9.2 | 116,180,404 | 106,626,177 | 79,757,598 |
| Control 4 | 8.6 | 145,750,150 | 138,011,408 | 103,118,946 |
| Control 5 | 8.8 | 136,358,786 | 130,146,156 | 103,218,996 |
| Control 6 | 8.7 | 118,492,534 | 115,638,465 | 96,997,166 |
| Control 7 | 7.9 | 91,686,360 | 89,344,952 | 71,384,926 |

| **Idiopathic gastroparetics** | | | | |
| --- | --- | --- | --- | --- |
|  | **RIN** | **Total reads** | **Mapped Reads** | **Gene Count** |
| Patient 1 | 7.9 | 119,484,062 | 115,425,708 | 89,093,292 |
| Patient 2 | 7.4 | 116,607,718 | 112,354,389 | 87,387,218 |
| Patient 3 | 9.1 | 115,655,728 | 111,148,280 | 83,064,334 |
| Patient 4 | 8.4 | 152,414,822 | 144,817,290 | 105,184,950 |
| Patient 5 | 9.4 | 134,842,202 | 126,102,956 | 94,537,748 |
| **Idiopathic controls** | | | | |
| Control 1 | 7.7 | 132,432,782 | 127,563,688 | 99,808,862 |
| Control 2 | 7.7 | 152,897,988 | 144,482,838 | 108,773,496 |
| Control 3 | 8.8 | 150,183,358 | 144,722,002 | 110,749,972 |
| Control 4 | 8.9 | 134,121,340 | 118,431,885 | 87,162,346 |
| Control 5 | 7.7 | 108,777,384 | 105,978,187 | 85,389,904 |
| Control 6 | 8.7 | 107,580,346 | 104,839,906 | 84,213,864 |
| Control 7 | 8.5 | 107,325,488 | 104,428,306 | 84,442,066 |
